# Bidi and Hookah Use Among Canadian Youth: Findings From the 2010 Canadian Youth Smoking Survey

**DOI:** 10.5888/pcd10.120290

**Published:** 2013-05-09

**Authors:** Christine D Czoli, Scott T Leatherdale, Vicki Rynard

**Affiliations:** Author Affiliations: Christine D. Czoli, Vicki Rynard, University of Waterloo, Waterloo, ON, Canada; PEER REVIEWED

## Abstract

**Introduction:**

Although cigarette use among Canadian youth has decreased significantly in recent years, alternative forms of tobacco use are becoming increasingly popular. Surveillance of youth tobacco use can help inform prevention programs by monitoring trends in risk behaviors. We examined the prevalence of bidi and hookah use and factors associated with their use among Canadian youth by using data from the 2010–2011 Youth Smoking Survey (YSS).

**Methods:**

We analyzed YSS data from 28,416 students (2006–2007) and 31,396 students (2010–2011) in grades 9 through 12 to examine prevalence of bidi and hookah use. We conducted multivariate logistic regression analyses of 2010–2011 YSS data to examine factors associated with bidi and hookah use.

**Results:**

From 2006 through 2010, prevalence of hookah use among Canadian youth increased by 6% (*P* = .02). Marijuana use emerged as a consistent predictor of bidi and hookah use. Males, youth of black, Latin, or other descent, and youth of Asian descent were more likely to use bidis (odds ratio [OR], 1.5; OR, 15.6; OR, 14.9) or hookah (OR, 1.3; OR, 2.4; OR, 1.5). Current cigarette smokers were more likely than nonsmokers to be current users of bidis (OR, 6.7) and hookahs (OR, 3.0), and occasional and frequent alcohol drinkers were also more likely than nondrinkers to be current hookah users (OR, 2.8; OR, 3.6).

**Conclusion:**

Although bidi use has not changed significantly among Canadian youth, the increase in hookah use warrants attention. Understanding the factors associated with use of bidis and hookahs can inform the development of tobacco use prevention programs to address emerging at-risk youth populations.

## Introduction

In recent years, patterns of global tobacco use among youth have changed substantially. Although cigarette use has decreased significantly, alternative forms of tobacco are becoming increasingly popular (1). In Canada, although cigarette smoking among youth decreased from 28.0% in 1999 to 12.0% in 2010 (2), data on the use of alternative tobacco products are limited. Many alternative tobacco products, including bidis and hookahs, pose significant health risks and sustain nicotine dependence (1,3,4). Furthermore, the popularity and perceptions of reduced harm associated with these products among youth and young adults have generated serious public health concern (5–7).

Bidi cigarettes contain tobacco hand-rolled in tendu leaf and look like hand-rolled marijuana cigarettes (4,8). According to the 2006–2007 Canadian Youth Smoking Survey (YSS) (8), 1.7% of Canadian youth had ever used bidis and 0.7% were current users. Sex, smoking status, marijuana use, alcohol use, race/ethnicity, level of education, exposure to peers who smoke, and depressive symptoms are associated with bidi use among youth and young adults (8–11).

Hookah, a water pipe that is used to smoke tobacco, originated in the Middle East (12). According to the 2006–2007 YSS, 6.8% of Canadian students reported ever using hookahs, and 2.7% were current users in 2006 (8). Existing evidence suggests that youth are more likely to use hookahs if they are male, smoke cigarettes, use marijuana or alcohol (8), know of a hookah lounge in the community, or believe that hookahs are more socially acceptable than cigarettes (13,14).

A better understanding of bidi and hookah use among youth is important for the development of future initiatives to prevent tobacco use. Ongoing surveillance of tobacco use among Canadian youth has not examined these issues since 2006 (8). We examined the prevalence of bidi and hookah use and associated factors among Canadian youth in 2010 and trends in prevalence since 2006.

## Methods

We used representative data collected from 31,396 Canadian students in grades 9 through 12 from 176 schools as part of the 2010–2011 YSS. The target population for the data used in this study consisted of all young Canadian residents in grades 9 through 12 attending public, private, and Catholic schools in 9 of the 10 Canadian provinces. Youth living in New Brunswick, Yukon, Nunavut, and the Northwest Territories were excluded from the target population, as were youth living in institutions or on First Nation Reserves and youth attending special schools or schools on military bases. The YSS was administered to students during class time, and participants were not compensated. The survey design and sample weights allow us to produce the population-based estimates in this article. Further details regarding recruitment and data collection for the YSS are available (15).

A combination of active information–passive permission and active permission protocols was used to recruit students. Active information–passive permission protocols required that parents call a toll-free number if they did not want their child to participate in the study after they had read an information letter describing the project, whereas active permission protocols required that students return a permission form signed by a parent or guardian. Students also had the opportunity to decline participation on the day of data collection. The response rate at the student level for the full 2010–2011 YSS (including students from grades 6 through 12) was 73.0% (15). The University of Waterloo Office of Research Ethics and appropriate school board and public health ethics committees approved all procedures, including passive consent.

Ever use of hookahs and bidis was measured by asking respondents to report if they had ever tried 1) using a water pipe to smoke tobacco (also known as a hookah, sheesha, narg-eelay, hubble-bubble, or gouza), or 2) smoking bidis (little cigarettes that are hand-rolled in leaves, tied with a string at the ends, and come in different flavors). Current use of hookahs and bidis was measured by asking respondents if in the last 30 days they had used 1) a water pipe to smoke tobacco (also known as a hookah, sheesha, narg-eelay, hubble-bubble, or gouza), or 2) bidis (little cigarettes that are hand-rolled in leaves, tied with a string at the ends, and come in different flavors).

Consistent with Health Canada’s definitions of smoking status for the YSS (16), smoking status was measured by asking respondents if they had ever smoked 100 or more whole cigarettes in their lifetime, and on how many of the last 30 days they smoked 1 or more cigarettes. Current smokers had smoked 100 cigarettes in their lifetime and had smoked in the previous 30 days; former smokers had smoked 100 cigarettes in their lifetime but had not smoked in the previous 30 days; and nonsmokers had smoked fewer than 100 cigarettes in their lifetime (15). The YSS also collected information on use of flavored tobacco products. Ever use of flavored tobacco products was measured by asking respondents if they had ever used flavored tobacco products (eg, menthol, cherry, strawberry, vanilla). Current use of flavored tobacco products was measured by asking respondents to check off the flavored tobacco products they had used in the last 30 days from a list (menthol cigarette, flavored cigarillo or little cigar, flavored cigar, flavored bidi, flavored smokeless tobacco, flavored tobacco in a water pipe). Marijuana use was assessed by asking respondents, “In the last 12 months, how often did you use marijuana or cannabis? (a joint, pot, weed, hash . . .)”. Those who reported never or not in the last 12 months were considered nonusers, those who reported using marijuana in the last year but not weekly were occasional users, and those who reported using marijuana weekly were considered frequent users. Alcohol use (binge drinking) was assessed by asking respondents, “In the last 12 months, how often did you have 5 drinks of alcohol or more on 1 occasion?” Those who reported never or not in the last 12 months were nonusers, those who reported binge drinking in the last year but not weekly were occasional drinkers, and those who reported binge drinking weekly were considered frequent drinkers. Demographic characteristics and further details regarding the 2010–2011 YSS questionnaire are available online (17).

Survey weights were used in all analyses to adjust for differential response rates across regions or groups and to adjust for complex sampling. Survey weights were developed in 2 stages. In the first stage, a weight (W1j) was created to account for the school selection within health region and school strata. In the second stage, another weight (W2jg) was calculated to adjust for student nonresponse. The weights were then calibrated to the provincial sex and grade distribution so that the total of the survey weights by sex, grade, and province would equal the actual enrollments in those groups. Descriptive analyses of the sample characteristics were examined by bidi ever use, bidi current use, hookah ever use, and hookah current use, using weighted data. Changes in prevalence of bidi and hookah ever and current use from 2006 through 2010 were also examined. We used data collected from 28,416 respondents in the 2006–2007 YSS, which was sampled, conducted, and weighted by using methods equivalent to those employed in 2010–2011. We then conducted 4 multivariate logistic regression models to examine factors associated with bidi ever use, hookah ever use, bidi current use, and hookah current use. SAS version 9.2 (SAS Institute Inc, Cary, North Carolina) was used for all analyses. Significance was set at *P* < .05.

## Results

Sample characteristics have been described previously (18). We calculated descriptive statistics for 2010 by examining bidi and hookah use among Canadian youth in grades 9 through 12 (Table 1), changes in the prevalence of bidi and hookah use from 2006 through 2010 (Table 2), and the results of logistic regression for factors associated with bidi and hookah use (Table 3).

**Table 1:** Characteristics of Youth in Grades 9–12, by Bidi and Hookah Use, Youth Smoking Survey, Canada, 2010^a^

Sample Characteristics	Prevalence (%)			
	Ever Use of Bidis (n = 1,601,400)^b^	Current Use of Bidis (n = 1,570,400)^b^	Ever Use of Hookahs (n = 1,601,400)^b^	Current Use of Hookahs (n = 1,570,400)^b^
**All Youth**	1.8	1.0	10.1	4.0
**Grade level**				
9th	1.7	1.1	5.4	2.2
10th	1.8	0.6	7.4	3.0
11th	2.1	1.2	12.0	4.6
12th	1.8	1.1	15.8	6.4
**Province^c^**				
Atlantic Canada^d^	2.9	1.6	6.8	3.5
Quebec	NA^f^	NA^f^	12.8	3.9
Ontario	1.1	0.6	8.7	4.1
Prairies^e^	2.7	1.7	10.3	3.6
British Columbia	1.9	1.1	11.9	4.6
**Race/ethnicity**				
White	1.3	0.4	10.1	3.6
Black, Latin, other	4.4	3.2	14.5	6.9
Asian	1.4	1.1	5.4	2.3
Aboriginal	2.4	1.8	9.4	4.5
**Cigarette smoking status^g^**				
Nonsmoker	0.9	0.4	6.4	2.1
Former	5.4	NA^f^	48.1	NA^f^
Current	11.1	6.6	40.3	21.2
**Ever use of flavored tobacco products^g^**				
No	0.3	0.2	2.4	0.7
Yes	6.1	3.2	32.1	13.8
**Current use of flavored bidi^g^**				
No	NA	0.3	9.2	3.2
Yes	NA	74.0	69.2	65.9
**Current use of flavored tobacco in a water pipe^g^**				
No	1.1	0.3	NA	1.5
Yes	25.0	21.3	NA	80.6
**Marijuana use status^g^**				
Nonuser	0.3	0.1	2.6	0.7
Occasional user	2.0	1.1	18.2	5.7
Frequent user	9.9	5.3	42.6	20.6
**Current alcohol use^g^**				
Nondrinker	0.4	0.2	2.0	0.6
Occasional drinker	1.4	0.4	12.0	4.3
Frequent drinker	6.8	4.2	29.8	13.2

Abbreviation: NA, not applicable.

a Analyses were conducted using weighted data.

b Weighted population estimates. Per Health Canada guidelines (16), weighted population estimates are rounded to the nearest hundred.

c It is illegal to sell or give tobacco to anyone under the age of 18 in the provinces of Alberta, Manitoba, Quebec, and Saskatchewan, and under the age of 19 in the provinces of British Columbia, Newfoundland and Labrador, Nova Scotia, Ontario, and Prince Edward Island.

d Atlantic Canada (Prince Edward Island, Nova Scotia, and Newfoundland and Labrador).

e Prairies (Alberta, Saskatchewan, and Manitoba).

f Data were suppressed because the unweighted sample size was less than 30.

g See Methods section for definitions of characteristics.

**Table 2:** Prevalence of Bidi and Hookah Use Among Youth in Grades 9–12, Youth Smoking Survey (YSS), Canada, 2006–2010^a^

Prevalence, %	2006	2010	% Change^b^	*P* Value^c^
**Ever use**				
**Total**	**n = 1,574,000^d^**	**n = 1,601,400^d^**	**NA**	**NA**
Bidi	2.2	1.8	−18	.002
Hookah	9.5	10.1	6	.02
**Female**	**n = 761,600^d^**	**n = 777,700^d^**	**NA**	**NA**
Bidi	1.5	1.0	−33	<.001
Hookah	7.7	8.5	10	.009
**Male**	**n = 812,400^d^**	**n = 823,700^d^**	**NA**	**NA**
Bidi	2.9	2.6	−10	.17
Hookah	11.2	11.6	4	.31
**Current use**				
**Total**	**n = 1,531,900^d^**	**n = 1,570,400^d^**	**NA**	**NA**
Bidi	0.8	1.0	25	.06
Hookah	3.9	4.0	3	.37
**Female**	**n = 742,400^d^**	**n = 766,800^d^**	**NA**	**NA**
Bidi	0.4	0.6	50	.007
Hookah	2.5	2.9	16	.02
**Male**	**n=789,500^d^**	**n = 803,700^d^**	**NA**	**NA**
Bidi	1.2	1.3	8	.57
Hookah	5.1	5.0	−2	.65

Abbreviations: NA, not applicable.

a Analyses were conducted using weighted data.

b Percentage change from 2006 through 2010 (percentage in 2006 minus the percentage in 2010 divided by the percentage in 2006).

c Calculated by using χ^2^ tests.

d Weighted population estimates. Per Health Canada guidelines, weighted population estimates are rounded to the nearest hundred.

**Table 3:** Multivariate Logistic Regression Analyses Examining Factors Associated With Bidi and Hookah Use Among Youth in Grades 9–12, Youth Smoking Survey, Canada, 2010^a^

Characteristic or Behavior	Model 1^b^		Model 2^c^		Model 3^d^		Model 4^e^	
	Ever Use of Bidis		Current Use of Bidis		Ever Use of Hookahs		Current Use of Hookahs	
	OR^f^ (95% CI)	*P* Value^g^	OR^f^ (95% CI)	*P* Value^g^	OR^f^ (95% CI)	*P* Value^g^	OR^f^ (95% CI)	*P* Value^g^
**Sex**								
Female	1 [Reference]							
Male	2.5 (2.0–3.1)	<.001	1.5 (1.1–2.1)	.01	1.1 (1.0–1.2)	.16	1.3 (1.1–1.4)	<.01
**Race/ethnicity**								
White	1 [Reference]							
Black, Latin, other	4.6 (3.7–5.8)	<.001	15.6 (10.8–22.5)	<.001	2.2 (1.9–2.5)	<.001	2.4 (2.1–2.9)	<.001
Asian	3.2 (2.2–4.7)	<.001	14.9 (8.9–24.9)	<.001	1.5 (1.3–1.8)	<.001	1.5 (1.2–2.0)	<.01
Aboriginal	1.1 (0.8–1.7)	.56	3.3 (1.9–5.7)	<.001	0.6 (0.5–0.7)	<.001	0.8 (0.6–1.1)	.20
**Cigarette smoking status^h^**								
Nonsmoker	1 [Reference]							
Former	1.9 (1.2–3.1)	.01	0.2 (0–0.7)	.02	5.5 (4.4–6.9)	<.001	3.3 (2.5–4.4)	<.001
Current	4.1 (3.3–5.2)	<.001	6.7 (4.8–9.5)	<.001	2.4 (2.1–2.6)	<.001	3.0 (2.5–3.5)	<.001
**Marijuana use status^h^**								
Nonuser	1 [Reference]							
Occasional user	4.9 (3.3–7.2)	<.001	23.9 (10.8–53.0)	<.001	4.8 (4.2–5.4)	<.001	5.6 (4.4–7.1)	<.001
Frequent user	13.1 (9.0–19.1)	<.001	50.4 (22.9–111.0)	<.001	11.6 (10.1–13.3)	<.001	14.1 (11.1–18.0)	<.001
**Current alcohol use status^h^**								
Nondrinker	1 [Reference]							
Occasional drinker	1.3 (0.9–2.0)	.16	0.4 (0.2–0.7)	<.01	2.5 (2.1–3.0)	<.001	2.8 (2.1–3.8)	<.001
Frequent drinker	2.2 (1.5–3.4)	<.001	1.3 (0.8–2.3)	.29	3.3 (2.8–4.0)	<.001	3.6 (2.7–5.0)	<.001

Abbreviations: OR, odds ratio; CI, confidence interval.

a Analyses were conducted using weighted data.

b Model 1: 1 = yes (n = 550), 0 = no (n = 27,123).

c Model 2: 1 = yes (n = 264), 0 = no (n = 26,937).

d Model 3: 1 = yes (n = 2,166), 0 = no (n = 25,507).

e Model 4: 1 = yes (n = 881), 0 = no (n = 26,320).

f Odds ratios adjusted for all other variables in the table and controlling for grade and province.

g Calculated by using Wald χ^2^ tests.

h See Methods section for definitions of characteristics.

### Ever use of bidis

Only 1.8% (n = 29,400) of Canadian youth in grades 9 through 12 in 2010 reported ever smoking bidis. Rates of bidi ever use were significantly higher in Atlantic Canada and the Prairies than in Ontario and Quebec (χ^2^ = 79.2, degrees of freedom (*df*) = 4, *P* < .001). Ever use of bidis was higher among respondents reporting ever use of flavored tobacco products (χ^2^ = 1,099.4, *df* = 1, *P* < .001) and among respondents who report current use of flavored tobacco in a water pipe (χ^2^ = 2,645.9, *df* = 1, *P* < .001). From 2006 through 2010, the prevalence of youth having ever smoked bidis decreased significantly by 18.2% and declined significantly more among female respondents (33.3%) than male respondents (10.3%).

As shown in Model 1 (Table 3), compared with female youth, male youth were more likely to have ever used bidis (odds ratio [OR], 2.5; 95% confidence interval [CI], 2.0–3.1). Compared with white respondents, students who self-identified as black, Latin, or other (OR, 4.6; 95% CI, 3.7–5.8) or Asian (OR, 3.2; 95% CI, 2.2–4.7) were more likely to have ever used bidis. Compared with nonsmokers, former cigarette smokers (OR, 1.9; 95% CI, 1.2–3.1) and current cigarette smokers (OR, 4.1; 95% CI, 3.3–5.2) were more likely to have ever used bidis. Occasional marijuana users (OR, 4.9; 95% CI, 3.3–7.2) and frequent marijuana users (OR, 13.1; 95% CI, 9.0–19.1) were more likely than nonusers to be ever users of bidis. Frequent alcohol drinkers were also more likely to be bidi ever users (OR, 2.2; 95% CI, 1.5–3.4) than were nondrinkers.

### Current use of bidis

Only 1.0% (n = 15,100) of Canadian youth in grades 9 through 12 in 2010 reported currently smoking bidis. Current bidi use was significantly lower in grade 10 than in grades 9, 11, and 12 (χ^2^ = 18.8, *df* = 3, *P* < .001). Rates of current bidi use were significantly higher in Atlantic Canada and the Prairies than in Ontario and Quebec (χ^2^ = 64.0, *df* = 4, *P* < .001). Current bidi use was higher among respondents reporting ever use of flavored tobacco products (χ^2^ = 578.1, *df* = 1, *P* < .001) and among respondents who reported current use of flavored tobacco in a water pipe (χ^2^ = 3,800.1, *df* = 1, *P* < .001). From 2006 through 2010, the prevalence of youth currently using bidis increased by 25.0% and increased substantially more among female (50.0%) than male (8.3%) respondents.

As shown in Model 2 (Table 3), compared with female youth, male youth were more likely to be current bidi users (OR, 1.5; 95% CI, 1.1–2.1). Compared with white respondents, those who self-identified as black, Latin, or other (OR, 15.6; 95% CI, 10.8–22.5), Asian (OR, 14.9; 95% CI, 8.9–24.9), or Aboriginal (OR, 3.3; 95% CI, 1.9–5.7) were more likely to be current bidi users. Former cigarette smokers were less likely than nonsmokers to be current bidi users (OR, 0.2; 95% CI, 0–0.7), whereas current cigarette smokers were more likely to be current bidi users (OR, 6.7; 95% CI, 4.8–9.5). Respondents who smoke marijuana occasionally (OR, 23.9; 95% CI, 10.8–53.0) or frequently (OR, 50.4; 95% CI, 22.9–111.0) were significantly more likely than marijuana nonsmokers to be current bidi users. Occasional alcohol drinkers were less likely to be current bidi users (OR, 0.4; 95% CI, 0.2–0.7) than were nondrinkers.

### Ever use of hookahs

In Canada, 10.1% (n = 161,300) of youth in grades 9 through 12 in 2010 reported ever using a hookah. Ever use of hookahs increased significantly by grade (χ^2^ = 535.9, *df* = 3, *P* < .001). Rates of hookah ever use were significantly higher in Quebec and British Columbia than in Atlantic Canada and Ontario (χ^2^ = 107.5, *df* = 4, *P* < .001). Ever use of hookah was higher among respondents reporting ever use of flavored tobacco products (χ^2^ = 5,642.0, *df* = 1, *P* < .001) and among respondents who reported current use of flavored bidis (χ^2 ^= 1,063.6, *df* = 1, *P* < .001). From 2006 through 2010, the prevalence of youth having ever smoked hookahs increased significantly by 6.3%, and a substantially larger increase was evident among female youth (10.4%) than male youth (3.6%).

As shown in Model 3 (Table 3), compared with whites, respondents who self-identified as black, Latin, or other (OR, 2.2; 95% CI, 1.9–2.5) or Asian (OR, 1.5; 95% CI, 1.3–1.8) were more likely to have ever used hookahs, whereas Aboriginal respondents were less likely to have ever used hookahs (OR, 0.6; 95% CI, 0.5–0.7). Former cigarette smokers (OR, 5.5; 95% CI, 4.4–6.9) and current cigarette smokers (OR, 2.4; 95% CI, 2.1–2.6) were more likely than nonsmokers to have ever used hookahs. Respondents who were occasional marijuana users (OR, 4.8; 95% CI, 4.2–5.4) and frequent marijuana users (OR, 11.6; 95% CI, 10.1–13.3) were more likely to be ever users of hookahs. Compared with nondrinkers, occasional alcohol drinkers (OR, 2.5; 95% CI, 2.1–3.0) and frequent alcohol drinkers (OR, 3.3; 95% CI, 2.8–4.0) were more likely to be ever users of hookahs.

### Current use of hookahs

In 2010, 4.0% (n = 62,900) of Canadian youth in grades 9 through 12 reported currently using hookahs. Current hookah use increased significantly by grade (χ^2^ = 194.0; *df* = 3, *P* < .001) and was higher among respondents reporting ever use of flavored tobacco products (χ^2^ = 2,495.7, *df* = 1, *P* < .001) and current use of flavored bidis (χ^2^ = 2,793.9, *df* = 1, *P* < .001). From 2006 through 2010, the prevalence of current hookah use increased by only 2.6%; the prevalence among female respondents increased significantly by 16.0%, whereas the prevalence among male respondents decreased by 2.0%.

As shown in Model 4 (Table 3), male youth were more likely than female youth to be current hookah users (OR, 1.3; 95% CI, 1.1–1.4). Compared with whites, respondents who self-identified as black, Latin, or other (OR, 2.4; 95% CI, 2.1–2.9) or Asian (OR, 1.5; 95% CI, 1.2–2.0) were more likely to be current hookah users. Compared with nonsmokers, former cigarette smokers (OR, 3.3; 95% CI, 2.5–4.4) and current cigarette smokers (OR, 3.0; 95% CI, 2.5–3.5) were more likely to be current hookah users. Respondents who smoke marijuana occasionally (OR, 5.6; 95% CI, 4.4–7.1) or frequently (OR, 14.1; 95% CI, 11.1–18.0) were significantly more likely than marijuana nonsmokers to be current hookah users. Compared with nondrinkers, occasional alcohol drinkers (OR, 2.8; 95% CI, 2.1–3.8) and frequent alcohol drinkers (OR, 3.6; 95% CI, 2.7–5.0) were more likely to be current hookah users.

## Discussion

From 2006 through 2010, the prevalence of both ever and current hookah use among Canadian youth increased. This finding is consistent with global trends marking the spread of hookah use among youth (12). Change in bidi use among Canadian youth from 2006 through 2010 was less concerning; ever use decreased significantly and current use increased modestly. Prevalence of current bidi use is similar among Canadian and American youth (9). Surveillance of such trends is critical to the development of tobacco control interventions that appropriately address the changing context of youth tobacco use patterns. Youth interventions should address the shift from cigarette use to use of alternative tobacco products; in the face of resource constraints, tobacco control efforts should focus on the rise in hookah use rather than use of bidis.

Marijuana use emerged as a consistent predictor of bidi and hookah use among Canadian youth. Occasional and frequent smokers of marijuana were more likely than nonsmokers to use or have used bidis or hookahs. These findings are consistent with existing research (8,10,19,20). Delnevo and Hrywna found a strong association between use of bidis and use of marijuana and blunts (cigars used for smoking marijuana by replacing some or all of the filler with marijuana), which may be explained by visual and perceived similarities between bidis and marijuana cigarettes (10). Indeed, in a study examining youth attitudes and beliefs toward alternative tobacco products, Soldz and Dorsey found that, among young people, the most endorsed attitude toward bidis was that they look like joints (21). This relationship could not be examined among Canadian youth using YSS measures. To adequately examine this association, measures that distinguish use of marijuana from use of blunts should be included in surveillance of tobacco use. In addition, given the recent decline in cigarette smoking prevalence and the clustering of alcohol, tobacco, and marijuana use among youth (22), the study findings offer further support for prevention programs to target multiple risk behaviors (23).

Examination of sociodemographic characteristics indicated patterns across race/ethnicity and sex for bidi and hookah use. Youth of Asian, black, Latin, or other race/ethnicity were more likely than white youth to report ever and current use of both bidis and hookahs, and youth of Aboriginal descent were more likely to be current users of bidis and ever users of hookahs than were white youth. Although black and Hispanic race/ethnicity have been associated with bidi use among youth in the United States (9,10), this finding is the first indication that an association between ethnicity and bidi use exists among Canadian youth. Although the association of black, Latin, or other race/ethnicity with bidi use mirrors that found in the United States, the association of Asian ethnicity and bidi use may be attributable to cultural traditions of Canada’s large South Asian population. The association of ethnicity with hookah use has been documented (8,13,19). Consistent with existing research (8–11,19,20), our study also found that male respondents were more likely than female respondents to report ever and current use of bidis and hookahs. Comparison of hookah use from 2006 through 2010 showed large and significant increases among female respondents. Given that such increases are similar to the uptake of cigarette smoking by US females in the 1960s (24), tobacco control strategies should address alternative tobacco products, and further research into sex differences in the use of these substances may be warranted.

We found that youth who are current or former smokers were more likely to report current use of bidis and hookahs than were nonsmokers. Occasional or frequent consumption of alcohol were also associated with current use of hookahs. Although these associations are consistent with the literature on co-occurrence of substance use (8–11,13,14,19), the exact nature of the relationship among these substances is not clear. Understanding this relationship requires further research to determine whether a particular substance serves as a gateway to other substance use or whether the substances are interrelated in some other way. Such research can be helpful to prevention programs targeting multiple risk behaviors (23). Longitudinal data are required.

Our study has limitations. First, the cross-sectional design of the study does not allow causal inferences to be drawn between use of bidis and hookahs and examined correlates or for the determination of temporality of use among the substances examined. Second, self-reported data derived from research examining substance use may be underreported. Third, the survey did not allow for the differentiation of methods pertaining to marijuana use, hindering the interpretation of associations between use of bidis and marijuana. Fourth, the study was limited to observations of co-occurrence of substance use because it examined risk of use rather than comorbid use of various substances. Finally, the analysis was limited to the representation of youth in mainstream school settings; youth who are not in school and youth who are in alternative school settings were excluded. This same limitation applies to the lack of representation of First Nation Peoples living on reserves.

The rise in hookah use warrants attention, given its increasing popularity. Efforts aimed at eliminating false perceptions of reduced harm associated with hookah use may show promise in reducing its social acceptability, especially as it becomes more prevalent. Factors associated with bidi and hookah use can help developers of prevention programs, and co-occurrence of substance use supports the development of an integrated approach to prevention that addresses multiple risk behaviors.
